# *In vivo* efficacy of enmetazobactam combined with cefepime in a murine pneumonia model induced by OXA-48-producing *Klebsiella pneumoniae*

**DOI:** 10.1128/spectrum.02345-24

**Published:** 2024-10-31

**Authors:** S. Albac, N. Anzala, P. Chavanet, N. Dunkel, J. Quevedo, A. Santerre Henriksen, D. Croisier

**Affiliations:** 1 Vivexia, Dijon, France; 2 Département d’Infectiologie, Centre Hospitalier Universitaire, Dijon, France; 3 Advanz Pharma, London, United Kingdom; 4 Maxel Consulting, Jyllinge, Denmark; Innovations Therapeutiques et Resistances (INTHERES), Toulouse, France

**Keywords:** pharmacokinetics, pharmacodynamics, enmetazobactam, cefepime, OXA-48, pneumonia model

## Abstract

Cefepime/enmetazobactam is a new β-lactam/β-lactamase inhibitor combination with broad-spectrum activity against multidrug-resistant Enterobacterales, including OXA-48-producing isolates. Furthermore, cefepime and enmetazobactam have demonstrated similar and excellent intrapulmonary penetration, supporting the use of this new antibiotic combination in the treatment of hospital-acquired pneumonia. This study evaluated the *in vivo* efficacy of cefepime/enmetazobactam in a murine neutropenic pneumonia model infected with various OXA-48-producing *K. pneumoniae* strains. Mice were subcutaneously administered with cefepime (100 mg/kg/q2h), alone or combined with enmetazobactam (30 mg/kg/q2h), or intraperitoneally with meropenem (100 mg/kg/q2h) at 2 h post-infection. Mice were euthanized at 26 h post-infection for bacterial enumeration in lungs and spleen. A robust growth was achieved in untreated control mice. Cefepime alone or meropenem had no effect on reducing the bacterial burden in lungs after a 24-h period of treatment. The addition of enmetazobactam to cefepime resulted in a 2-log_10_ CFU/g bioburden reduction in lungs compared to 26-h controls for all strains, including the strain harboring the highest MIC (= 8 µg/mL) to cefepime/enmetazobactam. When changes of bacterial burden were assessed relative to 2-h controls, bacterial stasis was observed. These data highlight the limited *in vivo* activity of meropenem against OXA-48-producing Enterobacterales despite *in vitro* susceptibility. Conversely, cefepime/enmetazobactam with a human-mimicking regimen demonstrated a significant antibacterial effect in the pneumonia model induced by three OXA-48-producing *K. pneumoniae* strains, compared with cefepime or meropenem at 24 h post-infection. Therefore, cefepime/enmetazobactam may be a new alternative for lung infections due to Enterobacterales producing OXA-48.

**IMPORTANCE:**

Third-generation cephalosporin-resistant *Klebsiella pneumoniae* with extended-spectrum β-lactamases as principal resistance determinants are classified as critical priority pathogens. Their increasing occurrence has led clinicians to widely use carbapenems. Accordingly, carbapenem resistance in *Klebsiella pneumoniae* has spread in recent decades across several countries, and OXA-48-like carbapenemases are one of the main determinants of carbapenem resistance in Enterobacterales. Cefepime/enmetazobactam is a novel β-lactam/β-lactamase inhibitor combination that demonstrated excellent intrapulmonary penetration, supporting its use in the treatment of pneumonia. This study examined the efficacy of enmetazobactam, in combination with cefepime, compared to carbapenems for OXA-48-producing *Klebsiella pneumoniae* in a 24-h murine neutropenic pneumonia model. The combination showed a bacteriostatic effect using the 2-h controls as reference. Compared to 24-h controls, and to cefepime or meropenem monotherapies, the co-administration of enmetazobactam with cefepime demonstrated a pronounced *in vivo* bactericidal activity against cefepime-non-susceptible *K. pneumoniae* isolates with cefepime/enmetazobactam MICs up to 8 µg/mL in this model.

## INTRODUCTION

*Klebsiella pneumoniae* is among the leading causes of healthcare-associated infections in hospitals globally and is primarily of concern in hospital-acquired pneumonia (HAP) or ventilator-associated pneumonia (VAP) in critically ill patients (1, 2). These infections are associated with significant mortality, and their treatment is challenging because of the current limited therapeutic options (3, 4). *K. pneumoniae* increasingly displays multidrug resistance mechanisms and readily acquires plasmid-mediated extended-spectrum β-lactamases (ESBLs), which represent the main determinants of third-generation cephalosporin resistance. Currently, cefotaxime (CTX)-M-type enzymes are the most commonly found ESBL type worldwide, and their increasing occurrence has led clinicians to widely use carbapenems, which are considered the last line in our therapeutic arsenal (5). Accordingly, carbapenem-resistant Enterobacterales (CRE) have spread in recent decades across several countries, and surveillance studies have shown that OXA-48-like carbapenemases are becoming the main determinants of carbapenem resistance in Enterobacterales, especially in European and Mediterranean countries (6–8). In this context, the World Health Organization has placed CRE as priority pathogens, for which new antibiotics and research are urgently needed (9).

Novel carbapenem-sparing approaches (as new combination of old agents or new agents) for empirical treatment of ESBL-producing Enterobacterales can represent a valuable option to limit the spread of carbapenem resistance (10). Enmetazobactam (ENM), formerly known as AAI101 (penicillanic acid sulfone), is a novel extended-spectrum β-lactamase inhibitor (BLI) with a unique mechanism that overcomes tazobactam-resistant variants of class A β-lactamases (11, 12). Cefepime (FEP) is a fourth-generation cephalosporin displaying an intrinsic activity against isolates expressing AmpCs and OXA-48-like carbapenemases.

The combination of enmetazobactam and cefepime exhibits potent *in vitro* and *in vivo* activity against isolates of Enterobacterales that coproduce class A and OXA-48 carbapenemases (13). In a few preclinical neutropenic thigh or lung infection models, enmetazobactam restored the efficacy of cefepime models against ESBL-producing Enterobacterales (14–16). In the neutropenic thigh infection models*,* the tested isolates covered a cefepime-enmetazobactam MIC range from 0.06 to >64 µg/mL. Depending on these previous publications, the estimated enmetazobactam *fT* (free time) > CT 2 µg/mL (CT = concentration threshold) was 8% to obtain a stasis and 44% to obtain a 1-log_10_ reduction in thigh bioburden (14, 16). In the pneumonia model, the tested isolates covered a cefepime-enmetazobactam MIC range from 0.06 to 2 µg/mL, and a 2-log_10_ kill in the lung was achieved with a plasma and epithelial lining fluid (ELF) cefepime *fT* > MIC of ≥20% and enmetazobactam *fT* >2 µg/mL of ≥20% of the dosing interval (15). However, today, no data are available on the efficacy of the combination ENM/FEP in the treatment of pneumonia induced by isolates having an MIC greater than 2 µg/mL.

The purpose of this study was to investigate the *in vivo* efficacy of cefepime/enmetazobactam compared to meropenem (MEM) against a panel of clinical OXA-48-producing *K. pneumoniae* in a neutropenic murine pneumonia model. These findings could help identify the optimal regimen for HAP/VAP treatment. (*Part of the data has been presented at the 2024 ESCMID Conference in Barcelona, Spain.*) (17)

## RESULTS

### MIC determination

The susceptibility of the three *K*. *pneumoniae* strains (*K.p 549*, *K.p 235,* and *K.p 246*) to meropenem and to cefepime alone or in the presence of enmetazobactam at a fixed concentration of 8 µg/mL was determined in triplicate. MICs to cefepime alone ranged from 16 to >256 µg/mL, indicating that all strains were resistant to cefepime (*R* >4 µg/mL according to the breakpoints from the EUCAST and *R* ≥16 µg/mL by CLSI/FDA breakpoints). The addition of enmetazobactam at a fixed concentration drastically reduced the MICs between 0.5 and 8 µg/mL for *K. pneumoniae* strains. MICs to meropenem ranged from 0.5 to >16 µg/mL. According to the breakpoints, only *K.p 549* was susceptible to meropenem with an MIC of 0.5 µg/mL (*S* ≤2 µg/mL for EUCAST and *S* ≤1 µg/mL for CLSI/FDA) (Table 1).

**TABLE 1:** Description of OXA-48/CTX-M-1- or CTX-M-15-producing strains of *K. pneumoniae* used in this study

Isolates	Resistance mechanism(s)	MIC (µg/mL)		
		Meropenem	Cefepime	Cefepime + enmetazobactam (8 µg/mL)
*K.p 549*	CTX-M-1; OXA-48	0.5	64	0.5
*K.p 235*	CTX-M-1; OXA-48	16	16	4
*K.p 246*	CTX-M-15; OXA-48	>16	>256	8

### Pharmacokinetic/pharmacodynamic analysis

**Fig 1:**
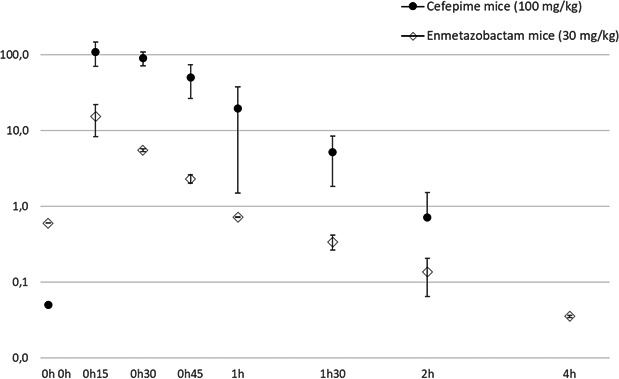
Plasma PK profile for cefepime and enmetazobactam; both drugs were administered separately subcutaneously (s.c.) at 0 h, with sampling over a 4-h period. A destructive design was used, with groups of mice at each time point being serially sacrificed. Data are means ± standard deviations from three mice.

The pharmacokinetics of cefepime (100 mg/kg) and enmetazobactam (30 mg/kg) were determined separately after a single subcutaneous injection over a 4-h period and are shown in Fig. 1. The total drug concentrations were determined. The plasma protein binding for enmetazobactam in mice being negligible, all concentrations were assumed to be 100% unbound (16). However, the protein binding for cefepime in mice is 10%–20% (18, 19).

The PK and the PK/PD parameters are shown in Tables 2 and 3, respectively.

**TABLE 2:** PK parameters (observed or estimated) from the concentration-time profiles for meropenem, cefepime, and enmetazobactam administered in mice^*a*^

Compound/ regimen	PK parameters			
	*t*_1/2_ (h)	*C*_max_ (µg/mL)	*C*_min_ (µg/mL)	AUC_0–24_ (µg·h/mL)
Meropenem IP				
100 mg/kg/**q2h** for 24 h (observed)	~ 15 min	59	0	303
Cefepime SC				
100 mg/kg/**q4h** for 24 h (observed)	~30 min	110	0	442
100 mg/kg/**q2h** for 24 h (estimated)	~30 min	110	0	876
Enmetazobactam SC				
30 mg/kg/**q4h** for 24 h (observed)	~15 min	15	0	39
30 mg/kg/**q2h** for 24 h (estimated)	~15 min	15	0	73

^*a*^ The values for meropenem PK presented in this table were obtained in a previous study (20).

**TABLE 3:** Simulated %*T* > MIC (or %*T* > fixed concentration) profile for meropenem, cefepime alone, enmetazobactam alone, and cefepime/enmetazobactam in mice receiving 100 mg/kg/q2h of meropenem interperitoneally, 100 mg/kg/q2h of cefepime, or/and 30 mg/kg/q2h of enmetazobactam subcutaneously

Strains	Estimated PK/PD parameters using an every 2-h dosing interval			
	MEM	FEP alone	ENM alone	FEP/ENM
	**%*T* > MIC**	**%*T* > MIC**	**%*T* > CT 2 µg/mL**	**%*T* > MIC**
*K.p 549*	74	25	37	100
*K.p 235*	12	50	37	75
*K.p 246*	5	0	37	62

The obtained PK parameters for the murine cefepime regimen (100 mg/kg/4 h) showed that the maximum antibiotic concentration achieved was *C*_max_ = 110 µg/mL, thus comparable to the one achieved in healthy volunteers receiving 2 g of cefepime every 8 h (*C*_max_ = 100 µg/mL on average) (21). However, the area under the concentration-time curve simulated in mice over a 24-h period (six injections every 4 h) was AUC_0–24_ = 442 µg·h/mL, thus approximately half the AUC_0–24_ observed in human (AUC_0–24_ = 800–900 µg·h/mL), suggesting that cefepime should be administered every 2 h in mice to achieve the human equivalent exposure.

The PK parameters for the enmetazobactam murine regimen (30 mg/kg/4 h) showed a *C*_max_ = 15 µg/mL on average, which was in-line with the data published by Bernhard et al. (14), using a 25 mg/kg IV dosage of enmetazobactam in mice and very close to the *C*_max_ (about 20 µg/mL) observed in healthy subjects with the approved dosing regimen (22). Enmetazobactam was undetectable at 4 h post-injection. The AUC_0–24_ simulated in mice over a 24-h period (six injections every 4 h) was AUC_0–24_ = 39 µg·h/mL, i.e., about half of the exposure observed in human (22), suggesting that enmetazobactam, similar to cefepime, should be administered every 2 h in mice to increase the percentage of time above the threshold.

These PK parameters were useful for the estimation of PK/PD criteria (%*T* > MIC) potentially associated with an antibacterial effect. The selected dose of 30 mg/kg of enmetazobactam produced a %*T* >2 µg/mL of 37% on average, using the q2h schedule.

Based on the pharmacokinetics of meropenem previously obtained in another study (20), a 100 mg/kg/q2h meropenem intraperitoneal regimen was associated to a 74%, 12%, and 5% of *T* > MIC for strain *K.p 549* (MIC = 0.5 µg/mL), *K.p 235* (MIC = 16 µg/mL), and *K.p 246* (MIC >16 µg/mL), respectively.

### *In vivo* efficacy in the neutropenic pneumonia mice model

**Fig 2:**
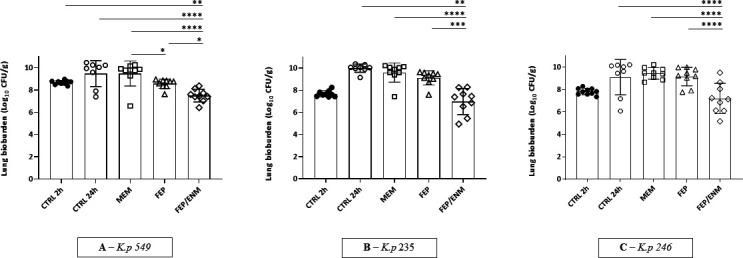
Therapeutic effects on bacterial load in lung (expressed as mean ± standard deviation of log_10_ of colony-forming units per gram of lung) after intranasal infection: comparison among three different *Klebsiella pneumoniae* strains producing OXA-48 and CTX-M1 or CTX-M15 (**A**: *K.p 549*; **B**: *K.p 235*; **C**: *K.p 246*). Quantitative variables were compared using an analysis of variance and a post hoc analysis using Bonferroni’s test. *P* < 0.05 was considered significant: *P < 0.05, **P < 0.01, ***P < 0.001, **** *P* < 0.0001. CTRL, control.

The antibacterial effect of cefepime combined with enmetazobactam was evaluated in the lung infection model; results are shown in Fig. 2 and in Table S1.

A robust infection was achieved in the vehicle group with all isolates, resulting in the following bioburdens in lungs at 26 h post-infection: 9.12 ± 1.6 log_10_ CFU/g for *K.p 235*, 9.4 ± 1.2 log_10_ CFU/g for *K.p 549*, and 10 ± 0.4 log_10_ CFU/g for *K.p 246*.

Meropenem administered at 100 mg/kg/24 h/q2h was ineffective in lungs, even in animals infected with the most susceptible strain (MIC = 0.5 µg/mL, 74% of *T* > MIC). As expected, same results were observed in animals receiving cefepime as their bacterial load in lungs essentially overlayed that from vehicle-treated controls.

The addition of enmetazobactam at 30 mg/kg to cefepime at 100 mg/kg/q2h for 24 h induced a significant bioburden reduction at 26 h in lungs (−2 log_10_ CFU/g on average), in all tested strains, even when mice were infected with strain *K.p 246*, which displayed the highest MIC (8 µg/mL) to the combination cefepime/enmetazobactam (−3 log_10_ CFU/g). When the bacterial burden in the lungs of treated animals (at 26 h) was compared to the bacterial burden in controls at 2 h (initiation of treatment), the magnitude of bacterial reduction was as follows: −0.92, −0.52, and −0.05 log_10_ CFU/g for *K.p 549*, *K.p 235,* and *K.p 246*, respectively (supplementary data S1), and stasis was demonstrated.

The antibacterial efficacy of all these regimens was also assessed in the spleen of infected animals and is presented in the Supplementary Data section. Based on the splenic data, no systemic translocation was observed at 2 h post-infection in control animals, while all of them were bacteremic at 26 h post-infection (5 log_10_ CFU/g on average). Surprisingly, all tested regimens, including cefepime and meropenem monotherapies, were associated with an antibacterial effect in the spleen, whatever the strains tested (supplementary data S2). This effect may be linked to the lower starting inoculum in the spleen compared to the lungs (23).

## DISCUSSION

The present study aimed at evaluating the efficacy of cefepime/enmetazobactam, a novel BL/BLI combination with broad-spectrum activity against multidrug-resistant Enterobacterales, in a neutropenic murine OXA-48 *K. pneumoniae* pneumonia model, using clinical isolates covering *in vitro* MIC values from 0.5 to 8 µg/mL. The obtained results demonstrated a clear added benefit of the combination enmetazobactam/cefepime over cefepime or meropenem monotherapy.

In this pneumonia model, a treatment with meropenem was unable to achieve any bacterial reduction in lungs, across the analysis of OXA-48-harboring isolates, compared to untreated animals at 26 h, and despite >70% *fT* > MIC obtained for the susceptible isolate (MIC = 0.5 µg/mL). This failure was not attributable to insufficient exposure because the same therapeutic regimen of meropenem (100 mg/kg/q2h) was tested in an ATCC 17978 *Acinetobacter baumannii* pneumonia model (MIC value 0.25 µg/mL to meropenem) and was associated with a massive bacterial reduction (−5 log_10_ CFU/g of lung) after treatment (24). Similar failures were depicted by others in different thigh and sepsis models due to *Klebsiella pneumoniae* producing the carbapenemase OXA-48, suggesting that carbapenems, even if active *in vitro* and efficient in the splenic compartment of our animals, may not be a reliable treatment for treating OXA-48 producers (25–27).

Conversely, a significant bacterial killing [range (−1.91, −3) Log_10_ CFU/g of lung] was recorded in mice receiving the cefepime/enmetazobactam regimen, when changes in bacterial burden relative to 26-h controls were assessed as the primary efficacy endpoint. This significant bacterial reduction was similar, whatever the tested strain, and even higher (−3 log_10_ CFU/g) for the strain harboring the highest MIC of the panel (8 µg/mL); these data provide the preclinical foundation of the efficacy of the cefepime/enmetazobactam combination on strains having MIC greater than 2 µg/mL. When the change of bacterial burden was assessed at 26 h relative to 2 h pretreatment time point, a bacterial stasis was demonstrated (at least <1 log_10_ CFU/g bacterial reduction, which is the standard preclinical surrogate for clinical efficacy in severe infections). Of note, the bacterial reduction was almost 1 log_10_ CFU/g (but not significant) for the most susceptible strain (MIC = 0.5 µg/mL).

A number of studies consistently identified fraction of the dosing interval above a free threshold concentration (%*fT* > CT) as the PK-PD index best describing the exposure-response relationship of enmetazobactam against ESBL-producing isolates of *K*. pneumoniae, followed by the *f*AUC > CT (15, 28, 29).

In a thigh model, Bernhard et al. showed that enmetazobactam exposures required for stasis and bioburden reduction (relative to the 2-h pretreatment time point) of 1 and 2 Δlog_10_ (CFU/g) were 12%, 27%, and 50% *fT* > CT 2 µg/mL, respectively (14). In a hollow-fiber model, Louie et al. demonstrated that an enmetazobactam PK-PD target of 31%–46% *fT* > CT 2 µg/mL was required for stasis to a 1-log_10_ bioburden reduction when combined with cefepime (28). In the mouse pneumonia model of Johnson et al., enmetazobactam plasma or epithelial lining fluid exposures of 20% *fT* > CT 2 µg/mL were required for a 2-log_10_ reduction in lung bioburden with a concomitant cefepime exposure of 20% *fT* > MIC, using strains with low MIC values to cefepime/enmetazobactam (0.06–2 µg/mL) (15). In our lung infection experimental conditions where a 37% *T* > CT 2 µg/mL (expressed as the total fraction, similar to the free fraction) was estimated in plasma using a q2h dosing regimen, a 2-log reduction in bioburden was recorded when compared to 26-h controls, and a stasis was obtained compared to pretreatment time point (2 h), at least for strains having an MIC of 4 or 8 µg/mL. This difference observed in terms of PK-PD target between the thigh model and the lung model may be due to the bacterial burden at the initiation of treatment, which can be much higher in the lung infection model (~7–8 log_10_ CFU/g) compared to the thigh model (~5–6 log_10_ CFU/g), thus impacting the bactericidal effect. In comparison, the *in vivo* data obtained in our model seem to fit well with the hollow fiber model where the inoculum is about 7 log_10_ at the start of treatment (29).

This difference could also be explained by the fact that the proportion of circulating antibiotic that reaches the pulmonary tissue compartment may be reduced. However, the pharmacokinetics of cefepime and enmetazobactam seem to have a highly predictable and linear behavior in mice (even infected), with similar partitioning into plasma and ELF (15). Clinical data highlighting similar concentration-time profiles of both agents in plasma and ELF have also been reported in healthy volunteers (21).

This study has limitations worth considering.

First, a limited number of isolates have been investigated in this preclinical study, even these three isolates span different levels of MICs to cefepime/enmetazobactam (covering a susceptibility range from 0.5 to 8 µg/mL). A larger number of isolates should be tested in this pneumonia model and probably more isolates displaying an MIC of 4 or 8 µg/mL, considering that by design, the conventional broth microdilution test has a onefold dilution error rate.

Second, the monotherapy regimens (meropenem and cefepime) mimicked the overall human exposure in terms of AUC_0–24_ but were not fully human-simulated ones (2 g q8h, 3 h infusion) and may not provide a best-case scenario for efficacy; they required a q2h schedule, which is not sustainable for repeated experimentation and raises significant ethical questions from an animal welfare perspective. The conservative dosing strategy applied in this study, based on pharmacokinetics data from non-infected animals, has most likely been improved in infected mice in relation to the pneumonia model, as described by others (30, 31).

Finally, even if this murine pneumonia model is very useful for early-stage studies, the short duration of treatment combined to the inoculum size at the initiation of treatment means the emergence of resistance cannot be explored. The U.S. Food and Drug Administration highlighted limitations of existing small animal models for evaluating antibacterial or prophylactic strategies against critical infections, such as HAP, caused by multidrug-resistant pathogens and asked for more diversified and bigger animal models to provide supportive data in the anticipation of clinical outcome and considering the recruitment of patients is highly challenging in such clinical situations (32). In particular, these complementary preclinical models should allow the administration of different dosing strategies and the adjustment of the drug exposure to a level similar to that anticipated in humans (often referred to as “humanized dosing”) to better anticipate the PK-PD indices. These models, such as the rabbit pneumonia models, are also expected to recapitulate more accurately the human disease, including similar clinical signs and high bacterial burden, useful to assess the risk of emergence of drug resistance (33).

Well-designed clinical studies assessing patient outcomes are warranted among patients infected with OXA-48-harboring isolates to assess the utility of carbapenems for these patients as this study confirmed the lack of *in vivo* efficacy in animal models as reported by others (26–28). Importantly, we need to clarify if the suggested enmetazobactam preclinical target of ~40% *fT* > CT 2 µg/mL, when administered in combination with high-dose cefepime, can represent a conservative estimate for use in clinical dose justification and breakpoint setting in the field of hospital-acquired bacterial pneumonia.

## MATERIALS AND METHODS

### Bacterial strains

Two CTX-M-1 and OXA-48- and one CTX-M-15 and OXA-48-producing *K. pneumoniae* clinical isolates were used in this study. *K. pneumoniae 2474549 (K.p 549*), *1865246* (*K.p 246*), and *1994235 (K.p 235*) were obtained from IHMA Europe Sàrl (Monthey, Switzerland), and bacterial stocks were kept at −80°C in cryobeads (bioMérieux, Marcy l’Etoile, France).

### MIC determination

The MICs were tested by twofold serial dilutions using the broth microdilution method according to the ISO 20776-1:2019 standards (34). Enmetazobactam was fixed at 8 µg/mL for cefepime/enmetazobactam MIC determination according to EUCAST recommendations (35). The inoculum suspension was adjusted in sterile water to a 0.5-McFarland suspension and subsequently diluted in cation-adjusted Mueller-Hinton Broth so that each well contained approximately 5 × 10^5^ CFU/mL. Antibiotic solutions were prepared following a dilution in Mueller-Hinton Broth of stock solutions stored at −80°C after reconstitution following manufacturer’s instructions. All microdilution plates were incubated at 37°C for 18 h in an ambient air incubator right after adding the inoculum, adjusting the final volume to 100 µL per well and careful sealing with a plastic film to prevent drying. Each MIC experiment was performed in triplicate.

### Animals and ethical aspects

A total of 147 CD1 mice (8–9 weeks old, 56–62 days) were used (Charles River, France) for the entire study. These animals were housed in a protected area at the animal facility of the University of Burgundy, Dijon, France (biosafety level 2 facility) and were fed *ad libitum* according to the current recommendations of the European Institute of Health. Before each experiment, animals were housed for 1 or 2 weeks at the animal facility. The animal facility was authorized by the French authorities (Agreement N° C 21 464 04 EA).

### Neutropenic murine lung infection model

Mice were immunosuppressed using cyclophosphamide (Baxter, France) at 150 mg/kg on day −4 and at 100 mg/kg on day −1 before bacterial inoculation (day 0). Mice were anesthetized with a mixture of isoflurane and oxygen, and intranasally instilled with 50 µL of a bacterial suspension containing 2 × 10^9^ CFU/mL.

### Pharmacokinetic/pharmacodynamic analysis

A pharmacokinetic study for meropenem was carried out as part of another study in mice receiving one intraperitoneal injection of meropenem (100 mg/kg) (20). The meropenem concentrations in plasma were determined in triplicate using a disk plate bioassay, with *Escherichia coli* NIJJHC2 as the indicator organism. The limit of detection was 0.2 µg/mL. Standard curves were established with solutions (progression from 0.5 to 10 µg/mL) in serum. The linearity of the standard curves used for disc plate bioassays was at least 0.97 (*r*^2^), and the mean coefficient of variation was 7%.

Uninfected mice received one subcutaneous injection of 100 mg/kg of cefepime or one injection of 30 mg/kg of enmetazobactam. Blood samples were collected at different time post-injection: 15 min, 30 min, 45 min, 1 h, 1 h 30 min, 2 h, and 4 h (*n* = 3 mice/time point). Concentrations of these antimicrobial agents were determined in cryopreserved plasma samples using an HPLC-MS/MS method (Laboratoire de Pharmacologie, CHU Amiens for cefepime and Charles River, Bioanalytics Department, Budapest, Hungary for enmetazobactam). The limit of detection, low limit of quantification, and upper limit of quantification values were 0.75, 2.5, and 369.6 µg/mL for FEP and 0.77, 0.77, and 1571.60 ng/mL for ENM.

For cefepime and enmetazobactam, the sampling strategy enabled the estimation of peak concentration (*C*_max_), minimal concentration total exposure (*C*_min_), area under plasma concentration vs time up to 24 h (AUC_0–24_), and half-life of elimination (*t*_1/2_). The percentage of time above the MIC (%*T* > MIC) was calculated as it is the most accurate (PK/PD) parameter predictive of efficacy for β-lactams. All PK and PK/PD parameters were modeled without correction for protein binding, based on negligible protein binding reported in mice and human for cefepime and for enmetazobactam (16).

### Animal treatment

At 2 h after inoculation, antibiotics were administered intraperitoneally (for meropenem and saline serum) or subcutaneously (for cefepime and enmetazobactam) and continued for 26 h. Animals were randomly assigned to one of four study arms: no treatment (saline serum), meropenem alone (100 mg/kg/q2h), cefepime alone (100 mg/kg/q2h), and cefepime (100 mg/kg/q2h) plus enmetazobactam (30 mg/kg/q2h). Nine mice per group and per isolate were culled at 26 h post-infection, and lungs and spleen were removed for bacterial counts. For animal receiving saline serum (controls), eight mice were also sacrificed 26 h post-infection. Organs were weighed, placed into a grinding tube containing a ceramic bead and 1 mL of saline PBS, and then homogenized in using the Fast Prep-24 5G (MP Biomedical, Fisher Scientific). Serial 10-fold dilutions of the homogenates were plated on Drigalski agar plates (bioMerieux, France) to determine the bacterial burden. Results were expressed in log_10_ CFU/g of organ.

### Statistical analysis

All data are expressed as mean ± standard deviation. For statistical comparisons of the differences between residual bacterial densities, culture-negative samples were considered to contain 1 log_10_ CFU/g. The statistical significance of differences between groups was determined by the one-way ANOVA followed by a post hoc Bonferroni test using Graph pad prism 9.0 (Graph Pad software, San Diego, CA).

## SUPPLEMENTAL MATERIAL

Supplemental material spectrum.02345-24-s0001.docx Tables S1 and S2; Fig. S1.

ASM does not own the copyrights to Supplemental Material that may be linked to, or accessed through, an article. The authors have granted ASM a non-exclusive, world-wide license to publish the Supplemental Material files. Please contact the corresponding author directly for reuse.
