# Supplementary material for: In vivo efficacy of enmetazobactam combined with cefepime in a murine pneumonia model induced by OXA-48-producing Klebsiella pneumoniae
Source: Microbiol Spectr. 2024 Oct 31;12(12):e02345-24. doi: 10.1128/spectrum.02345-24 (PMC11619402; doi:10.1128/spectrum.02345-24)

**SD 1**. Pulmonary bioburden (expressed as mean ± standard deviation of Log_10_ of colony-forming units per gram of lung) obtained after intranasal infection with different *Klebsiella pneumoniae* strains producing OXA-48 and CTX-M-1 or CTX-M-15. MEM: meropenem, FEP: cefepime; ENM: enmetazobactam.

| **Therapeutic**  **regimen** | ***K.p 549 Strain*** | | ***K.p 235 Strain*** | | ***K.p 246 Strain*** | |
| --- | --- | --- | --- | --- | --- | --- |
|  | 2h | 26h | 2h | 26h | 2h | 26h |
| Control | 8.7 ± 0.2 | 9.5 ± 1.2 | 7.9 ± 0.2 | 9.1 ± 1.6 | 7.7 ± 0.3 | 9.9 ± 0.3 |
| MEM | - | 9.5 ± 1.1 | - | 9.4 ± 0.5 | - | 9.6 ± 0.9 |
| FEP | - | 8.6 ± 0.4 | - | 9.2 ± 0.8 | - | 9.1 ± 0.6 |
| FEP/ENM | - | 7.7 ± 0.6 | - | 7.2 ± 1.3 | - | 6.99 ± 1.2 |

**SD2**. Splenic bioburden (expressed as mean ± standard deviation of Log_10_ of colony-forming units per gram of lung) obtained after intranasal infection with different *Klebsiella pneumoniae* strains producing OXA-48 and CTX-M-1 or CTX-M-15. MEM: meropenem, FEP: cefepime; ENM: enmetazobactam.

| **Therapeutic**  **regimen** | ***K.p 549 Strain*** | | ***K.p 235 Strain*** | | ***K.p 246 Strain*** | |
| --- | --- | --- | --- | --- | --- | --- |
|  | 2h | 26h | 2h | 26h | 2h | 26h |
| Control | 1.4 ± 0.7 | 5.0 ± 1.0 | 3.3 ± 0.5 | 6.2 ± 0.9 | 1.2 ± 0.5 | 4.5 ± 1.0 |
| MEM | - | 1.2 ± 0.5 | - | 3.4 ± 1.1 | - | 3.3 ± 1.3 |
| FEP | - | 1.2 ± 0.5 | - | 1.5 ± 0.8 | - | 1.65 ± 0.9 |
| FEP/ENM | - | 1.0 ± 0.0 | - | 1.3 ± 0.6 | - | 1.3 ± 0.5 |

**SD1**. Therapeutic effects on bacterial load in spleen (expressed as mean ± standard deviation of Log_10_ of colony-forming units per gram of lung) after intranasal infection: comparison among three different *Klebsiella pneumoniae* strains producing OXA-48 and CTX-M1 or CTX-M15 (**A**: *K.p 549*; **B**: *K.p 235*; **C**: *K.p 246*).

Quantitative variables were compared using an analysis of variance and a post-hoc analysis using Bonferroni’s test. *p*< 0.05 was considered significant: * *p*<0.05, ** *p*<0.01, *** *p*<0.001, **** *p*<0.0001. CTRL: Control, FEP: cefepime; ENM: enmetazobactam, MEM: meropenem.


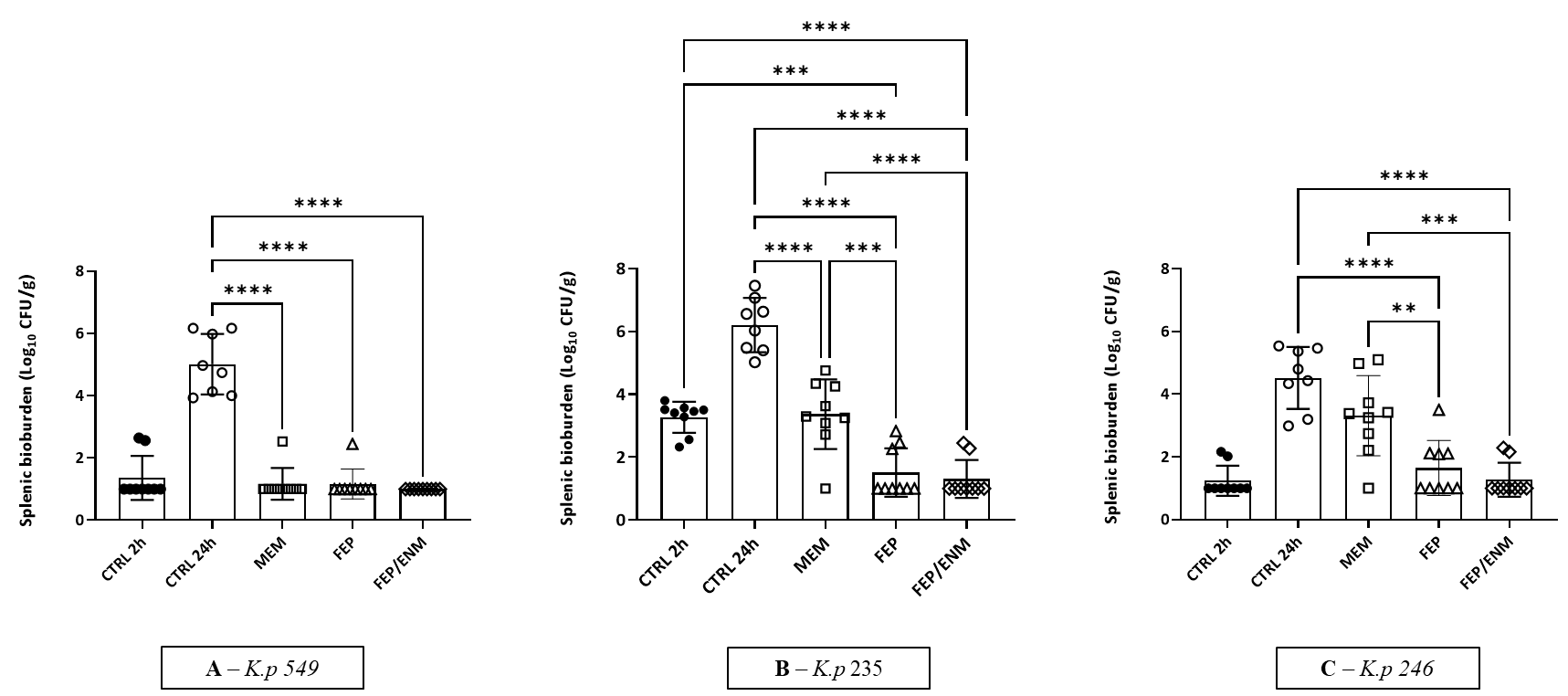

Supplement: Supplemental material — Tables S1 and S2; Fig. S1. [file spectrum.02345-24-s0001.docx]
